# Sterility in the offspring of *spr-5; met-2* mutants may be caused by inherited H3K4 methylation and altered germline transcription

**DOI:** 10.17912/micropub.biology.001365

**Published:** 2024-10-05

**Authors:** Jazmin Dozier, Mattie Villhauer, Brandon Carpenter

**Affiliations:** 1 Molecular and Cellular Biology, Kennesaw State University, Kennesaw, Georgia, United States

## Abstract

During maternal reprogramming of histone methylation in *C. elegans* , H3K4me is removed by the histone demethylase, SPR-5 , and H3K9me is subsequently added by the histone methyltransferase, MET-2 . Maternal loss of SPR-5 and MET-2 causes inherited phenotypes, such as sterility, in the progeny. Here, we find that knocking down either the H3K4 methyltransferase SET-2 or the H3K36 methyltransferase MES-4 partially rescues the germline in the progeny of *spr-5 ; met-2* mutants, suggesting that the inherited sterility may be caused by inherited H3K4 methylation and altered germline transcription.

**Figure 1. Knocking down SET-2 and MES-4 partially rescues the germline in spr-5; met-2 progeny:**
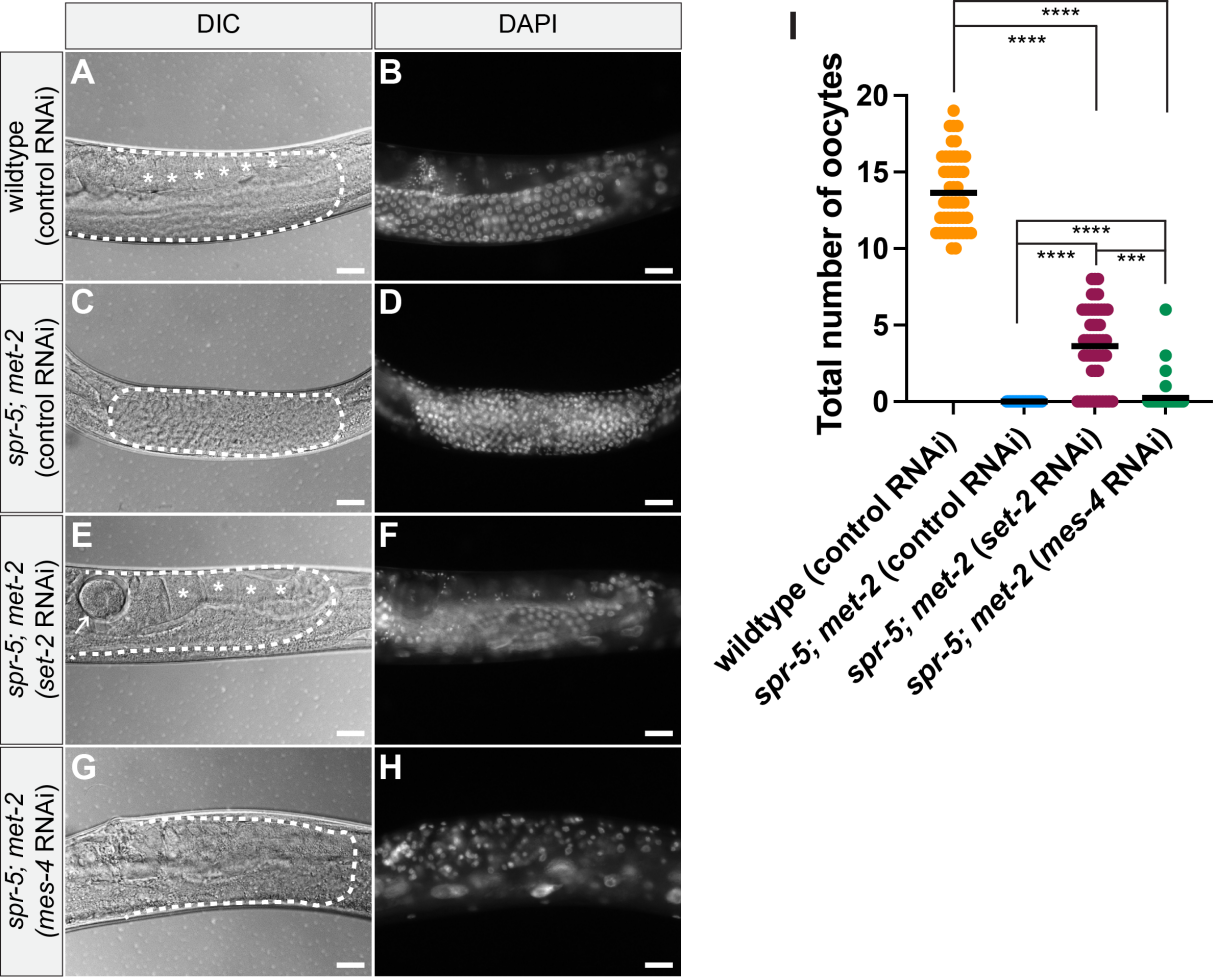
40x Differential Interference Contrast (DIC) (A, C, E G) and DAPI (B, D, F, H) images of wildtype ( N2 strain) (A-B), or *spr-5 ; met-2* (C-H) progeny from hermaphrodite parents fed control (empty vector L4440) RNAi (A-D), *set-2* RNAi (E-F), or *mes-4* RNAi (G-H) three days (wildtype fed control RNAi) (A-B), four days ( *spr-5 ; met-2* fed *set-2* or *mes-4* RNAi) (E-H), and five days ( *spr-5 ; met-2* fed control RNAi) post hatching (C-D) when progeny reached young adulthood. Dashed lines outline the posterior gonad arm (A-B, E-H) or the entire germline (C-D). Asterisks (*) denote oocytes. Arrow denotes an embryo. Scale Bar: 20µm. (I) Quantification of the total number of oocytes counted across both gonad arms from wildtype progeny on control RNAi (N=48) and *spr-5 ; met-2* progeny on either control (N=50), *set-2* RNAi (N=50), or *mes-4* RNAi (N=50) (unpaired t-test, **** represent a p-value <0.0001, *** represent a p-value <0.05).

## Description

Chemical modifications to the N-terminal tails of histone core proteins regulate gene expression by controlling accessibility of the transcriptional machinery to the DNA (Burton and Torres-Padilla, 2014; Hirabayashi and Gotoh, 2010; Jambhekar et al., 2019) . For example, methylation of lysine 4 or 36 on histone 3 (H3K4me and H3K36me) is generally associated with accessible chromatin and active transcription, whereas methylation of lysine 9 and 27 (H3K9me and H3K27me) on histone 3 is associated with closed chromatin and repressed transcription (Bannister and Kouzarides, 2005; Barski et al., 2007; Bernstein et al., 2002; Bernstein et al., 2005) . Histone methylation can also be inherited through mitotic divisions and across generations where it plays critical roles in establishing developmental cell fates, including germline versus somatic cell fates (Carpenter et al., 2021; Gaydos et al., 2014; Jambhekar et al., 2019; Kaneshiro et al., 2019; Katz et al., 2009; Öst et al., 2014; Siklenka et al., 2015; Tabuchi et al., 2018).

During gametogenesis in *Caenorhabditis elegans* ( *C. elegans* ), the COMPASS Complex, which includes the methyltransferase, SET-2 , adds H3K4me1/2 at germline genes and is required for normal germline identity and germline gene transcription (Li and Kelly, 2011; Robert et al., 2014; Simonet et al., 2007; Xiao et al., 2011) . However, at fertilization, this H3K4me1/2 must be erased, or maternally reprogrammed, to properly establish germline versus somatic cell fates in the subsequent embryo. During maternal reprogramming of histone methylation in *C. elegans* , H3K4me1/2 is removed by the H3K4 demethylase, SPR-5 , and H3K9me1/2 is subsequently added by the histone methyltransferase, MET-2 (Carpenter et al., 2021; Katz et al., 2009; Kerr et al., 2014) . Progeny of mutants lacking both SPR-5 and MET-2 are developmentally delayed and completely sterile in a single generation while displaying synergistic increases in both H3K4me2 and germline gene expression (Kerr et al., 2014; Carpenter et al., 2021) .

In addition to erasing histone methylation, some inherited histone methylation must be actively maintained between generations to re-establish germ cell fate during early embryogenesis. To do this in *C. elegans* , the H3K36me2/3 methyltransferase, MES-4 , is deposited into the oocyte where at fertilization it maintains H3K36me2/3 at a subset of germline genes that were expressed in the parental germline (hereafter referred to as MES-4 germline genes) (Furuhashi et al., 2010; Rechtsteiner et al., 2010) . Maternal loss of MES-4 causes improper germline proliferation in the progeny but does not affect normal somatic development (Capowski et al., 1991; Garvin et al., 1998) .

Our previous work demonstrates that knocking down either SET-2 or MES-4 rescues developmental delay in *spr-5 ; met-2* mutant progeny suggesting that ectopic accumulation of H3K4me1/2 and H3K36me2/3 contributes to the developmental delay in *spr-5 ; met-2* mutant progeny (Carpenter et al., 2021) . However, whether inherited H3K4me1/2 and H3K36me2/3 also affects sterility in *spr-5 ; met-2* mutant progeny was not examined. Here, we test this possibility by feeding *spr-5 ; met-2* mutant hermaphrodites either *set-2* or *mes-4* RNAi and examining germlines of their synchronized progeny at the young adult stage using DIC microscopy, DAPI staining, and by quantifying the total number of oocytes across both gonad arms ( **Figure 1** ). At 72 hours post hatching, wildtype germlines on control RNAi are properly elongated with normally stained germ line cells, sperm, and an average of 13.7 oocytes across both gonad arms ( **Figure 1A, B, and I** ). Consistent with previous work that characterized *spr-5 ; met-2* mutant adult germlines, *spr-5 ; met-2* progeny on control RNAi displayed unelongated and highly disorganized germlines consisting of mostly undifferentiated germline progenitor cells and sperm (Kerr et al., 2014) , **Figure 1B and 1C** ). No oocytes were detected out of 50 analyzed germlines ( **Figure 1I** ). To directly test whether inappropriately inherited H3K4me1/2 is contributing to sterility in the next generation, we examined germlines of *spr-5 ; met-2* progeny on *set-2* RNAi and observed that these germlines were more elongated and organized compared to progeny from *spr-5 ; met-2* mutants fed control RNAi ( **Figure 1E and 1F** ). Additionally, these germlines had an average number of 3.6 oocytes across both gonad arms, with 19 out of 50 containing at least five oocytes and 11 out of 50 of these germlines containing no oocytes ( **Figure 1I** ). We also observed the presence of embryos in some of the germlines of *spr-5 ; met-2* mutants fed *set-2* RNAi ( **Figure 1E** , arrow).

We next tested whether it is the inappropriate germline transcription that is contributing to sterility in *spr-5 ; met-2* progeny. Despite the requirement for MES-4 for normal germline formation (Capowski et al., 1991; Garvin et al., 1998) , progeny of *spr-5 ; met-2* mutants fed *mes-4* RNAi displayed healthier, more elongated germlines largely composed of undifferentiated germ cells ( **Figure 1G and 1H** ). Unlike the germlines of *spr-5 ; met-2* mutants fed *set-2* RNAi, germlines of *spr-5 ; met-2* progeny on *mes-4* RNAi lacked embryos and only 4 out of the 50 germlines scored contained oocytes ( **Figure 1I** ).

The data presented here demonstrate that knocking down the histone methyltransferases SET-2 and MES-4 partially rescues germline health in *spr-5 ; met-2* progeny. Our current working model is that in the absence of SPR-5 ; MET-2 maternal reprogramming, inappropriately inherited H3K4me1/2 facilitates aberrant H3K36me2/3 mediated transcription by maintaining a chromatin environment that is more permissive than normal. Since there is a tightly controlled sequence of germline transcription required for normal germline proliferation, spermatogenesis, and oogenesis, any untimely germline gene expression is likely to perturb germline formation and cause sterility. Our previous work demonstrated that the somatic developmental delay in *spr-5 ; met-2* mutant progeny depends on the somatic expression of MES-4 germline genes (Carpenter et al., 2021) . It is possible that the unusually permissive chromatin environment created by loss of SPR-5 ; MET-2 maternal reprogramming may interfere with the normal progression of the germline by causing certain MES-4 germline genes to be expressed in the germline at the wrong levels or the wrong time. Alternatively, aberrant expression of other germline genes, or somatic genes, in the *spr-5 ; met-2* germline could contribute to sterility in *spr-5 ; met-2* germlines. For example, *C. elegans* lacking SPR-5 in addition to the chromatin remodeler LET-418 /Mi2 display germlines that phenocopy *spr-5 ; met-2* germlines. In these *spr-5 , let-418* germlines, the germ cells lose their pluripotency and reprogram into neurons (Käser-Pébernard et al., 2013) . Future transcriptional analysis on *spr-5 ; met-2* germlines and *spr-5 ; met-2* germlines rescued by knocking down SET-2 and MES-4 will help decipher between these possibilities. Because *C. elegans* lacking MES-4 are completely sterile and contain germlines that fail to proliferate (Capowski et al., 1991; Garvin et al., 1998) , we did not expect germlines to form at all when we knocked down MES-4 . The finding that knocking down MES-4 rescues *spr-5 ; met-2* mutant germlines suggest that there is a certain chromatin level required for proper germline transcription and knocking down MES-4 restores the balance. However, neither knock down of SET-2 nor MES-4 were able to completely rescue the germline health back to wildtype levels. These data suggest that reducing the inappropriate levels of either H3K4me1/2 or H3K36me2/3 is not enough to completely restore the balance of the chromatin environment back to normal.

Lastly, the lack of gonad arm extension suggests that defects in distal tip cells (DTCs) may also contribute to sterility in *spr-5 ; met-2* mutants. Thus, it is possible that suppression of sterility phenotype following *mes-4* and *set-2* RNAi may in part be due to suppression of defects in DTCs, which are required for normal gonad arm extension. However, additional experiments are required to characterize the DTC defects in *spr-5 ; met-2* mutants and the suppression of sterility following *mes-4* and *set-2* RNAi. While the mechanisms underlying sterility in *spr-5 ; met-2* mutants remain to be determined, our results provide further evidence that *C. elegans* utilizes a combination of inherited histone modifications to achieve the correct levels of transcription to properly specify tissues.

## Methods

***C. elegans Strains***

All *C. elegans* strains were grown and maintained at 20°C. The *C. elegans* *spr-5 ( by101 ) / tmC27 [ unc-75 ( tmls1239 )] (I)* ; *met-2 ( n4256 )] / qc1 (III)* strain (Carpenter et al., 2021) and the wildtype, ( N2 ) strain were provided by the Katz Lab (Emory University, GA, Emory).

***RNAi Interference***

RNA interference (RNAi) experiments were performed as previously described (Carpenter et al., 2021) . In brief, F0 worms were placed on RNAi plates (NGM plates containing 100 μg/ml ampicillin, 0.4 mM IPTG, and 12.5 μg/ml tetracycline) as L3 larvae, and then moved to fresh RNAi plates approximately 48 hours later where they were allowed to lay embryos for 2-4 hours to synchronize F1 progeny. F1 progeny were then DAPI stained and DIC imaged as young adults. Some *spr-5 ; met-2* progeny develops to young adults five days after a synchronized lay (Carpenter et al., 2021) . We were also able to obtain higher numbers of young adult *spr-5 ; met-2* mutant progeny on either *set-2* or *mes-4* RNAi four days after a synchronized lay. Thus, to ensure that we analyzed stage matched young adult germlines across all genotypes and RNAi treatments we examined germlines of wildtype progeny on control RNAi at three days, *spr-5 ; met-2* progeny on either *set-2* or *mes-4* RNAi at four days, and *spr-5 ; met-2* progeny on control RNAi at five days post synchronized lay. For each RNAi experiment reported here, *pos-1* RNAi resulted in 100% embryonic lethality, indicating RNAi plates were optimal.

***DAPI Staining and Differential Interference Contrast Imaging***

The 4′,6-diamidino-2-phenylindolea (DAPI) staining technique used was previously described (Cecchetelli et al., 2016) to observe oocytes and sperm within gonad arms. Worm strains were DAPI stained at the young adult stage of development. All worms were washed using phosphate buffered saline and 0.1% Tween 20 (PBST) in a nine-well spot plate, and then fixed in cold methanol for 20 minutes. After fixation, the worms were washed again in PBST, and stained with DAPI (100 ng/ml) in PBST for at least 20 minutes. The worms were then put on a slide coated with 2% agarose pad in M9 buffer. Posterior gonad arms of the germline were Differential Interference Contrast (DIC) imaged at 40X magnification using an Olympus BX61 epifluorescence microscope equipped with a DAPI florescent filter.
